# Evaluation of water quality and soil fertility in remediated farmland for protection of wetland ecology by planting different crops

**DOI:** 10.7717/peerj.20406

**Published:** 2025-11-28

**Authors:** Changqing Liu, Zhongxiang Sun, Hongyang Wang, Tianwen Chen, Lina Deng, Li Zhang, Huixing Liang, Yuxiang Shen, Hongshan Li, Hai Cheng

**Affiliations:** 1 Yancheng Institute of Technology, Yancheng, Jiangsu, China; 2 Yancheng Polytechnic College, Jiangsu Yancheng, China; 3 Jiangsu Yancheng National Rare Bird Nature Reserve Management Office, Jiangsu Yancheng, China

**Keywords:** Remediated farmland, Wetland, Water quality, Soil fertility, Migratory birds

## Abstract

Enhancements in water quality and soil characteristics of wetlands can improve the ecological environment of the area and enrich its biodiversity. The present study examined the effects of five distinct crops (*i.e.*: colza oil, mustard, Chinese cabbage, wheat, barley) cultivated in remediation plots, and evaluated their impact on water quality and soil fertility. The water quality within the remediated farmland was categorized as Class III (moderately polluted). The soil exhibited low total nitrogen and organic matter levels (the mean values were respectively 0.032% and 10.84 g/kg), and high readily available phosphorus and potassium concentration (the mean values were respectively 75.71 and 247.64 mg/kg). The soil fertility was comprehensively categorized as Class III (moderately polluted). Subsequently, the different components of bird droppings in the soil were investigated for their potential effects on soil fertility. The present research demonstrated that the remediation of farmland had the potential to enhance the quality of water and soil fertility in wetland. This, in turn, might result in an increased number of migratory birds inhabiting the area.

## Introduction

Wetland ecosystems provide ecological services, such as purification of water, degradation of pollutants and protection of species that help sustain ecological equilibrium through the resident microorganisms, aquatic organisms, plants, and animals (Gu & Wu, 2023; Rowland, Hagger, and Lovelock, 2023; Zhi et al., 2023). Globally, wetland areas have shrunk by more than 35% due to degradation and destruction caused by both climate change and human activities, including industrialization, urbanization and tourism development (Wang et al., 2024; Fedyń et al., 2023; Ferrarini, Celada & Gustin, 2021). The Yancheng coastal wetland is populated by salt-tolerant plants (*Suaeda salsa*) and provides habitat for migratory birds (*Grus japonensis*) and elk. However, the wetland is also affected by species invasions, agricultural development, and human activities. There is an urgent need to restore the wetland to protect the habitat of rare birds and to promote biodiversity in the area (Wang et al., 2021a; McNeil et al., 2020).

A growing number of researchers are focusing on wetland conservation. They are proposing many methods of wetland remediation. The excessive growth of reeds in remediated wetlands may reduce desirable wetland functions. Researchers measured the growth differences of reeds in response to flooding, mowing, and their combinations. The results showed that moderate flooding combined with treatment could effectively inhibit the excessive growth of reeds (Park et al., 2024). The habitat of Chongming Dongtan Wetland was optimized by implementing the semi-closed reclamation project, which also controlled the invasion of *Spartina alterniflora*, an invasive saltmarsh grass. In addition, a macro-benthic ecological survey revealed that the macro-benthic density and biomass in the ecological enhancement project were significantly lower than those in the natural tidal flat (Huang et al., 2022). The remediation of coastal wetland had been used to create mosaic-type habitats that attracted more birds, improved biodiversity and provided a reference for remediating degraded coastal ecosystems (He et al., 2023). Yancheng Wetland is divided into a core area and a buffer area, the latter being larger and more affected by human activities, particularly agricultural expansion. To improve the migratory bird habitat in the degraded buffer zone of Yancheng Wetland, ecological remediation should be carried out (Ferreira et al., 2023; Barik, Saha & Mazumda, 2022).

The early human activities that have been observed in this region have resulted in the continuous expansion of wetland buffer zones for cultivable farmland (Levitt, Lai & Manville, 2022). Farmers have historically adopted pesticides and fertilizers with the aim of increasing farmland productivity (Williams, Borjesson & Hedlund, 2013). This has resulted in changes to the soil composition and water quality. The inland salt marshes were affected by degradation and soil desertification, necessitating research on wetland remediation strategies to improve the soil quality of these marshes. By using remote sensing and wetland investigation methods, the differences in soil properties of different remediation stages of wetlands were investigated. The results showed that the remediation period had a significant impact on the total phosphorus content of the soil (Zhao et al., 2024b). The implementation of the Floodplain Ecological Remediation Project has led to the partial recovery of water quality, sediment and organisms in the area (Roni et al., 2019). Consequently, the efficacy of wetland remediation initiatives in enhancing water quality and augmenting plant diversity in affected regions is indisputable. Moreover, these endeavors contribute to the establishment of a conducive habitat for migratory birds and other fauna, thereby enhancing the local ecological conditions.

The remediation of wetland farmland has been demonstrated to result in an improvement in regional soil quality, including soil fertility, soil organic matter, and others (Lu et al., 2024a; Lu et al., 2024b; Wu et al., 2023). In the scientific assessment of soil quality with six different methods for sustainable vegetation eco-remediation in engineered disturbed areas, Zhao et al. (2024a) evaluated the soil quality using the soil quality index and found that the vegetation concrete ecological remediation slope (VC) and frame beam fill soil slope (FB) methods had good remediation effects and were conducive to ecological remediation. In order to restore the functionality of the reservoir inundation regions’ soil and ecological environment, the Vietnamese government conducted studies which monitored topography, slope, inundation time, soil type and water quality. These studies provided a theoretical foundation for subsequent remediation projects (Quang et al., 2022). Consequently, it is imperative to consider the alteration of soil properties during wetland farmland remediation to ensure the provision of nutrient-rich soil in the area.

The Yancheng wetland serves as a transitional zone between the warm temperate zone and the northern subtropical zone, which is mainly influenced by a marine and continental climate, characterized by suitable temperature, abundant rainfall, and sufficient light (Duan et al., 2020; Li et al., 2020). Every year, the wetland attracts a large number of migratory birds, including several endangered and rare species such as *Grus japonensis*, *Ciconia boyciana*, *Eurynorhynchus pygmaus*, and *Larus saundersi*, which reside and winter in Yancheng (Wang et al., 2020b; Wang et al., 2019). Due to its comprehensive ecological system, robust ecological regulation, and high habitat value (Wang et al., 2020a), Yancheng Wetland has been recognized on the World Heritage List: Migratory Bird Sanctuaries along the Coast of the Yellow Sea-Bohai Gulf of China (Phase I) beginning in 2019 (https://whc.unesco.org/en/list/1606/). The ecological environment of the peripheral buffer zone of the Yancheng Wetlands has been degraded by urbanization, agricultural expansion and tourism development. In addition, the soil fertility has been depleted, and water quality has deteriorated, affecting the ability of waterfowl to forage and reducing the area of a suitable habitat. Therefore, it is imperative that a restored habitat is implemented to improve soil fertility and water quality, safeguard the ecological environment and promote biodiversity in the area.

The authors aim to study soil and water quality characteristics to simultaneously strengthen wetland farm ecosystems and promote the diversity of migratory birds by planting multiple crops on remediated farmland. The present study analyzed various water quality parameters in fields cultivated with different crops during different periods. The investigation further sought to ascertain the impact of migratory bird droppings on the soil parameters within the wetland farmland environment. The authors conducted a comparative analysis of various physical and chemical soil properties in fields where different crops were cultivated. The objective of the research is to compare soil and water quality parameters under different remediation treatment methods, with the aim of improving the remediation results of wetland farmland in the protected area. The overarching objective of the program is to increase the number and diversity of migratory birds.

## Materials and Metheds

### Study area

The study site is located in within the southern buffer region of the Yancheng coastal wetland, which includes an area of agricultural reclamation between 33°30′N–33°32′N latitude and 120°30′E–120°33′E longitude (Fig. S1).

### Experimental design

**Figure 1:**
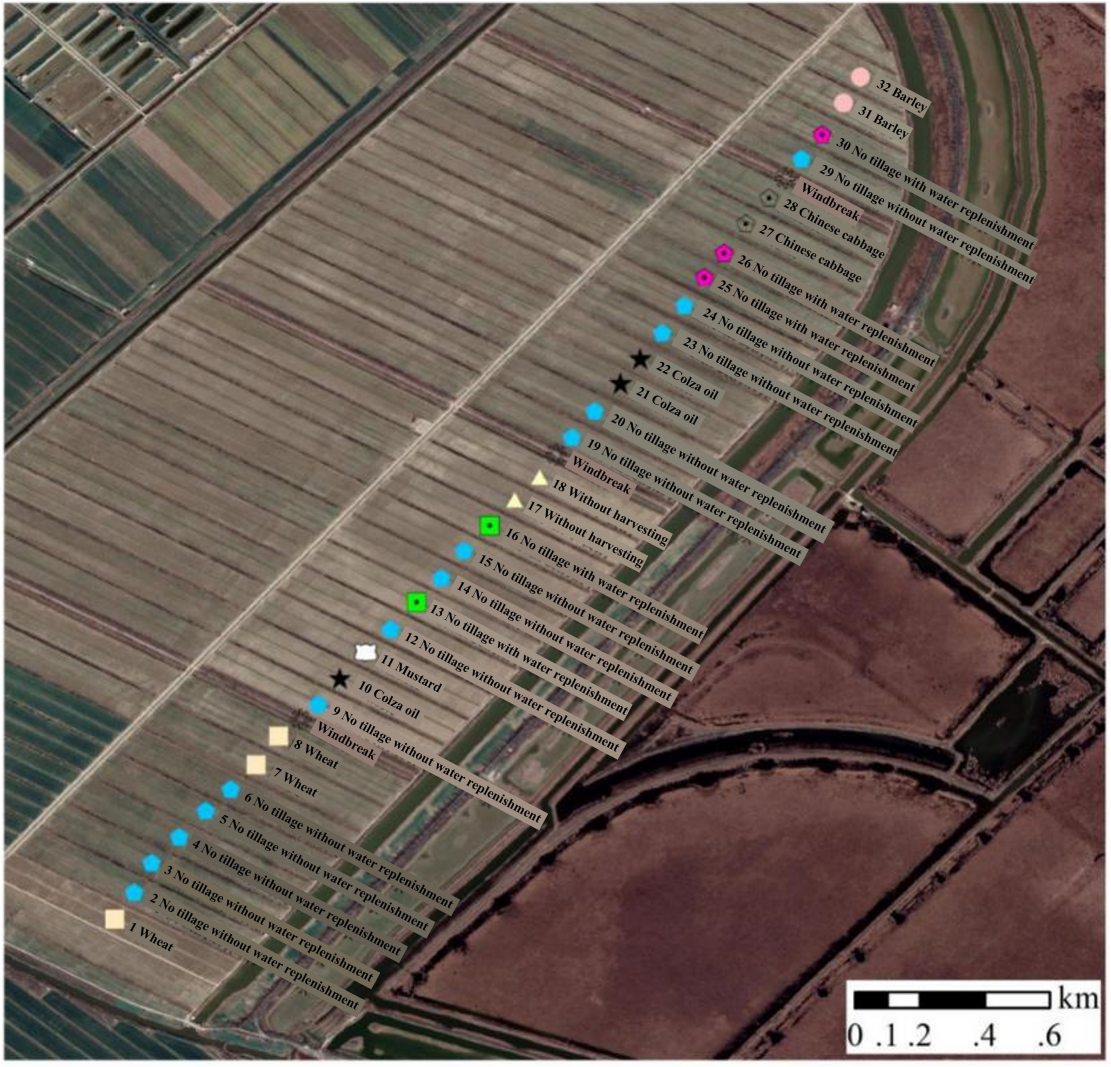
The plot allocation of wetland farmland remediation area.

Based on previous relevant studies, we adopted a randomized block design and conducted different treatments after rice harvest to explore the effects of different remediated farmland on rare birds and water quality of the soil (Liu et al., 2024). A total of eight experiments were conducted, comprising the following treatments: sowing colza oil, sowing mustard, sowing Chinese cabbage, sowing wheat, and sowing barley after rice harvesting, respectively, artificial straw crushing, used as a mulch, after rice harvesting, and no-tillage after rice ripening (with water replenishment, without water replenishment). The plot allocation of wetland farmland remediation area was displayed in Fig. 1. A total of eight experimental plots were sampled as illustrated in the Table 1.

**Table 1:** The water quality monitoring and statistics of single factor results in dry season (sulfide: mg/L; mercury, arsenic, lead, cadmium: μg/L). C28: Chinese cabbage; C1: Wheat; C31: Barley; C10: Colza oil; C11: Mustard; C6: No tillage without water replenishment; C25: No tillage with water replenishment; C17: Without harvesting.

Area site	Sulfide	Mercury	Arsenic	Lead	Cadmium
C28	<0.005	<0.04	<0.3	<1.0	<0.1
C1	<0.005	<0.04	<0.3	<1.0	<0.1
C31	<0.005	<0.04	<0.3	<1.0	<0.1
C10	<0.005	<0.04	<0.3	<1.0	<0.1
C11	<0.005	<0.04	<0.3	<1.0	<0.1
C6	<0.005	<0.04	<0.3	<1.0	<0.1
C25	<0.005	<0.04	<0.3	<1.0	<0.1
C17	<0.005	<0.04	<0.3	<1.0	<0.1

 The researchers sampled the soil in the eight treatment plots and randomly selected two surrounding plots as controls. They used the 5-point sampling method consisting of three layers (0–20 cm, 20–40 cm, 40–60 cm). In each plot, five random sampling points were established in a plum blossom formation, and samples were taken using a Φ70 mm stainless steel soil drill. After sampling, samples from the same layer were combined to form representative soil samples for each area. The researchers collected bird droppings from eight different treated sites and pooled bird droppings form each site as a single sample.

Water samples were collected from the outfalls and various drainage ditches within the rice field remediation project area. Samples were analyzed and monitored twice in late February (dry season) and late May (normal season). The processing carried out for each plot was as follows: C28: Chinese cabbage; C1: Wheat; C31: Barley; C10: Colza oil; C11: Mustard; C6: No tillage without water replenishment; C25: No tillage with water replenishment; C17: Without harvesting.

### Observations of birds

During clear days (*i.e.,* excluding periods of heavy rain, fog or strong winds), the presence or singing of species was recorded at both the experimental site (the remediated region) and the control site (the plot where wheat was planted after rice harvest) by two experienced observers. The foraging period of the birds in this region was primarily concentrated during the overwintering period, which extended from late November to February of the subsequent year.

Table S1 provides a synopsis of the numerical data and taxonomic classification of avian species observed at various locations and temporal points. The authors conducted data analysis and found that the species and numbers of birds in the farmland remediation area were higher than those in the control area. For detailed data analysis and result comparisons, please refer to Liu’s paper (Liu et al., 2024).

### Determination of water quality parameters

The total nitrogen (TN) concentration was determined by means of an alkaline potassium persulfate digestion process, followed by ultraviolet spectrophotometry (wavelength 210 nm). The quantification of total phosphorus (TP) was conducted through the utilization of an alkaline potassium persulfate digestion method, complemented by molybdenum-antimony anti-colour spectrophotometry (at a wavelength of 700 nm). Potassium permanganate was determined by acidification and oxidation and then measured by ultraviolet spectrophotometry at 525 nm. The determination of BOD_5_ was accomplished through the implementation of the iodimetry and dichromate method. The mass of total suspended solids was measured by drying at 105 °C (Zhang et al., 2024). The total salt content of the water samples was conducted to ascertain their total salt content, following a process of evaporation. The presence of sulphur compounds present in water was detected by methylene blue spectrophotometry at a wavelength of 660 nm (Sad et al., 2015). The mercury, arsenic, lead, and cadmium in water were determined by means of graphite furnace atomic absorption spectrophotometry. The pH values were measured using a pH meter (Li et al., 2024).

### Determination of soil fertility parameters

Soil samples and bird droppings were collected and subsequently dried in an indoor environment to eliminate any extraneous contaminants. Subsequently, larger particles were subjected to grinding and sieving. The droppings were meticulously extracted from the soil, debris, and foliage, and subsequently transferred into a ziplock bag for low-temperature storage in order to facilitate the detection of indicators such as organic matter, total nitrogen, total phosphorus, and total potassium.

The organic matter content was determined by employing potassium dichromate volumetric method and external heating methods (Anwar et al., 2024). The Kjeldahl method with a continuous flow analyzer (Bran and Luebbe TRAACS Model 2000 Analyzer) was employed to determine total nitrogen, while spectrophotometry with potassium persulfate oxidation molybdenum blue colorimetric method was utilized for total phosphorus in both soil and bird droppings using colorimetry (Hou et al., 2021). The alkali diffusion method was utilized to determine the amount of alkali hydrolyzed nitrogen. The sodium bicarbonate method was utilized to measure available phosphorus in real time. The hydrofluoric acid perchloric acid method was utilized to estimate total potassium. The ammonium acetate extraction method was utilized for the expeditious estimation of available potassium (Yang et al., 2022). The alkali hydrolyzed nitrogen, available phosphorus, total potassium, total N and available potassium were determined by elemental analyzer (Elementar; Vario Macro, Langenselbold, Germany). The organic matter, total nitrogen, readily available phosphorus and readily available potassium were selected as the parameters for the comprehensive evaluation and analysis of the soil. The values (*Wi*) of each parameter were the weight of the *i* index, *Fi* was the score value of the *i* index, and *I* was the comprehensive fertility index of the plot. These parameters were selected as the criteria for evaluating the comprehensive characteristics of the soil because they account for a large proportion in the soil and have a significant impact.

### Evaluation method

#### Comprehensive pollution index of water quality

The values of four factors, total nitrogen, total phosphorus, potassium permanganate index, and BOD_5_, were inputted into the Nemerow pollution index equation to calculate the comprehensive pollution index *p*-value.

\begin{eqnarray*}p=\sqrt{ \frac{({p}_{i}^{2}+{p}_{imax}^{2})}{2} } \end{eqnarray*}

where: *p*_*i*_ was the average single pollution index, ${p}_{i}= \frac{1}{n} \sum \frac{{C}_{i}}{{S}_{i}} $, *C*_*i*_ was measured value, *S*_*i*_ was standard values; *p*_*imax*_ was the maximum single index among all the pollution factors.

#### Soil fertility by bird droppings accumulating

A large amount of bird droppings could accumulate on the soil surface, which might change soil fertility. The improvement in soil fertility could be estimated using the following formula:

\begin{eqnarray*}M=B\ast N\ast W\ast t\ast \frac{C}{1000\ast 1000} \end{eqnarray*}

where: *M* was the increased value of a fertility index (kg/hm^2^); *B* was the number of birds feeding on the plot each day; *N* was the average number of defecations per day; *W* was the dry weight per defecation (g); *t* was the time spent foraging on the plot (d); *C* was the content (%) of a fertility index in bird droppings. 1,000 was the area of the plot (hm^2^). The model was modified from the models used to estimate Tundra Swan droppings (Somura et al., 2015).

#### Comprehensive fertility and soil fertility grade

The following additive model was used to calculate and evaluate the comprehensive fertility index of the plot, and the soil fertility grade was evaluated according to the value *I*.

\begin{eqnarray*}I=\sum {F}_{i}\ast {W}_{i}(i=1,2,3,\ldots \ldots ,n) \end{eqnarray*}

where *I* was the comprehensive fertility index of the plot, *F*_i_ was the score value of the *i* index, and *W*_i_ was the weight of the *i* index.

### Statistical analyses

All data obtained in shake flask culture process were the mean of triplicate experiments. The statistical significances of differences in chemical and physical properties of water quality, soil and bird droppings were evaluated using a one-way analysis of variance (ANOVA) and Duncan’s multiple range tests in SPSS version 16.0. A value of *P* < 0.05 was considered statistically significant.

**Figure 2:**
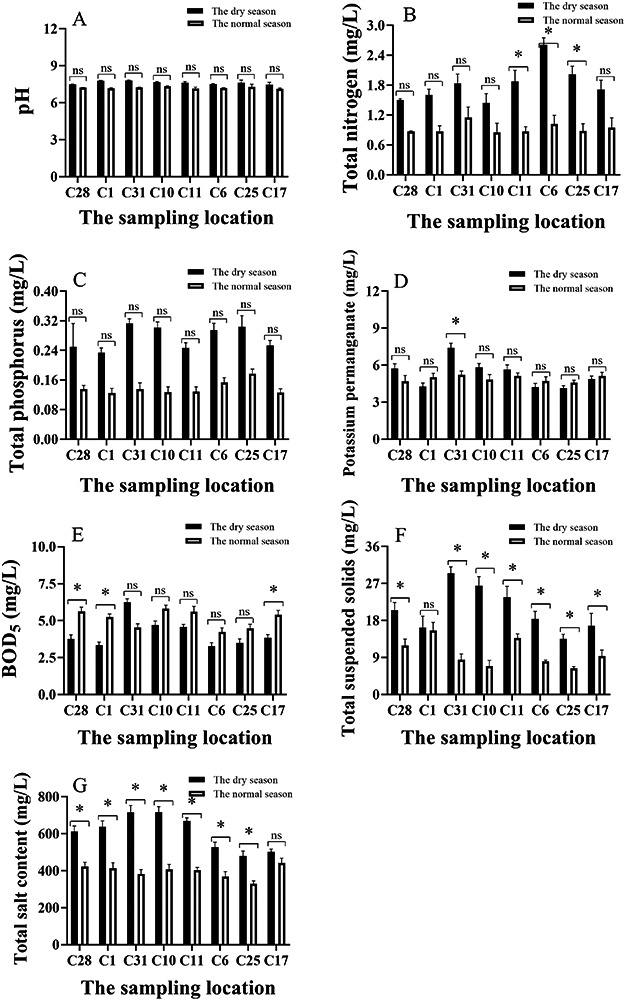
(A–G) Analysis of main physical and chemical characteristics of water in different season. C28: Chinese cabbage; C1: Wheat; C31: Barley; C10: Colza oil; C11: Mustard; C6: No tillage without water replenishment; C25: No tillage with water replenishment; C17: Without harvesting. *: *p* ≤ 0.05; ns: *p* > 0.05.

## Results

### Effects of wetland farmland remediation project on water quality

A total of eight distinct categories of remediation sites were selected for water quality measurement, with the objective of conducting a comparative analysis across different seasons, namely the dry and normal seasons. The pH of the water ranged from 7.18 to 7.83 in Fig. 2A, which was classified as being weakly alkaline. Furthermore, a small increase in pH value was observed during the dry period when compared to the normal period. The mean concentration of total nitrogen during the dry period was found to be greater than 1.50 mg/L, with a maximum recorded 2.65 mg/L. Also, the content of total nitrogen during dry season in C11, C6 and C25 was significantly higher than that during normal season (*p* < 0.05). As demonstrated in Fig. 2B, the mean content of total nitrogen during the normal period was 0.97 mg/L. The total phosphorus content during the dry period exhibited a higher value (0.29 mg/L) in comparison to the normal period (0.14 mg/L). The maximum value recorded was 0.31mg/L, observed at sample point C31, as illustrated in Fig. 2C.

The permanganate index and the biological oxygen demand (BOD_5_) was utilized to detect organic and inorganic oxidizable matter pollution in water. The mean average value of the potassium permanganate index during the dry and normal periods was 5.54 and 4.98 mg/L, respectively. The maximum value recorded was 7.1 mg/L in C31, in which the concentration of potassium permanganate during the dry period was significantly higher than that during normal season (*p* < 0.05, see Fig. 2D). It was observed that the BOD_5_ at a concentration of 5.12 mg/L during the normal period was marginally higher than that the concentration of 4.15 mg/L during the dry period. The maximum BOD_5_ concentration recorded during the dry period was 6.24 mg/L (Fig. 2E). The content of BOD_5_ during dry season in C28, C1 and C17 was significantly higher than that during normal season (*p* < 0.05).

Suspended solids were also indicative of turbidity levels in the water, with higher concentration recorded during the dry period (22.24 mg/L). The mean concentration of suspended matter during the normal period was found to be significantly lower than that during the dry season except C1 (*p* < 0.05), with an average of 9.31 mg/L as illustrated in Fig. 2F. The mean concentration of total salt during the dry period (660 mg/L) significantly exceeded that of the normal period (393 mg/L) without C17 (*p* < 0.05, Fig. 2G). The sulphide content of the water in the wetland farmland remediation project area was found to be minimal, with concentration consistently below 0.005 mg/L during both dry and normal periods. The remediation project area exhibited minimal concentrations of heavy metals in the water with mercury, arsenic, lead and cadmium not detected during both dry and normal periods (Tables 1 and 2).

### Analysis of bird droppings in the wetland farmland remediation project area

**Figure 3:**
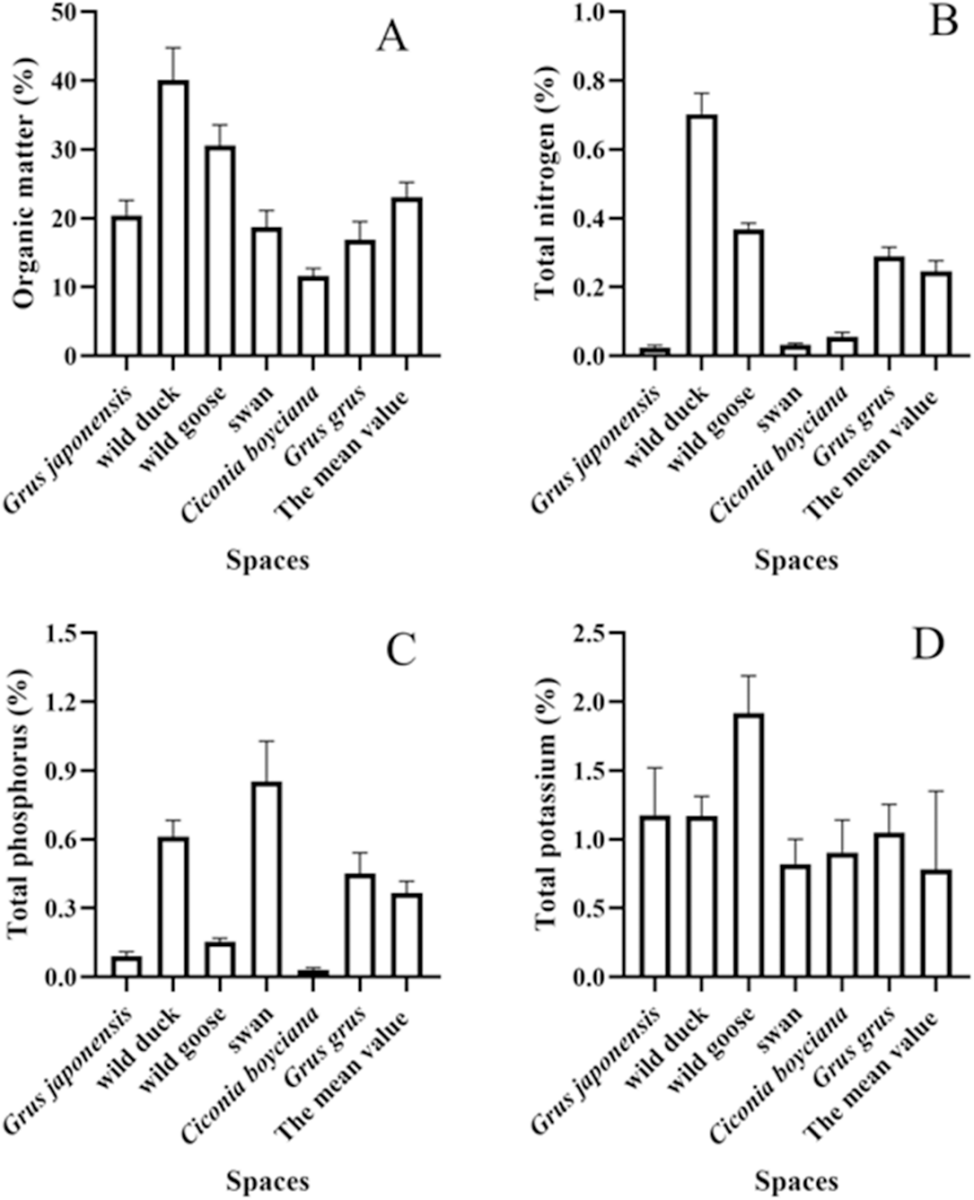
(A–D) Analysis of nutrient composition of bird droppings. *: *P* ≤ 0.05; ns: *P* > 0.05.

Bird droppings, which were obtained from six species in the farmland plots: *Grus japonensis*, wild duck, wild goose, swan, *Ciconia boyciana* and *Grus grus*. The content of organic matter in the six collected types of bird droppings ranged from 11.60% to 40.12%, with an average of 23.07%. The concentration of organic matter in wild duck and wild goose was found to be 40.12% and 30.61%. Furthermore, the contents of total nitrogen, total phosphorus and total potassium in bird droppings, respectively, ranged from 0.025% to 0.703%, 0.032% to 0.852%, and 0.822% to 1.918%, respectively, and the corresponding average values were 0.246%, 0.366% and 1.173% in Fig. 3.

**Table 2:** The water quality monitoring and statistics of single factor results in normal season (sulfide: mg/L; mercury, arsenic, lead, cadmium: μg/L). C28: Chinese cabbage; C1: Wheat; C31: Barley; C10: Colza oil; C11: Mustard; C6: No tillage without water replenishment; C25: No tillage with water replenishment; C17: Without harvesting.

Area site	Sulfide	Mercury	Arsenic	Lead	Cadmium
C28	<0.005	<0.04	0.66	<1.0	<0.1
C1	<0.005	<0.04	3.89	<1.0	<0.1
C31	<0.005	<0.04	1.35	<1.0	<0.1
C10	<0.005	<0.04	7.12	<1.0	<0.1
C11	<0.005	<0.04	3.25	<1.0	<0.1
C6	<0.005	<0.04	7.12	<1.0	<0.1
C25	<0.005	<0.04	4.71	<1.0	<0.1
C17	<0.005	<0.04	4.01	<1.0	<0.1

### Effects of wetland farmland remediation project on soil fertility

#### pH and organic matter of soil

The pH value of the soil in the various plots of the wetland farmland remediation project is presented in Table 3. The value of the treatment group was between 7.62–9.19, and that of the control group was between 8.60–8.96. All of the values were apparently alkaline, in accordance with the characteristics of the saline alkaline soil in this area. The range of soil organic matter in the various plots of the remediation project area was found to be between 1.14 and 18.80 g/kg, the mean value was 10.84 g/kg. The concentration of soil organic matter in the control area was found to be between 13.8 and 17.0 g/kg, the mean value was 15.13 g/kg. The Second National Soil Survey, in conjunction with the six-level classification standard of soil nutrients in China, indicated that the organic matter content in this area was at a low level comparing the mean values with the experimental group and control group. This finding was consistent with the characteristics of low organic matter in the coastal saline alkaline soil of Jiangsu.

**Table 3:** Basic physical and chemical properties of soil fertility in eight different fields in the wetland farmland remediation project.

Area	No.	Total potassium/(%)		Quick available potassium/(mg/kg)		Total phosphorus/ (%)		Quick available phosphorus/ (mg/kg)		Total nitrogen /(%)	Alkali- hydrolyzed nitrogen /(mg/kg)	Organic matter /(g/kg)	pH
		K	K_2_O	K	K_2_O	P	P_2_O_5_	P	P_2_O_5_				
C1	C1- surface	0.84	1.01	166.4	300.57	0.087	0.198	71.56	163.90	0.052	49.80	11.20	8.65
	C1- middle	0.86	1.04	126.2	352.13	0.074	0.169	33.52	76.77	0.040	49.80	18.80	8.83
	C1- bottom	0.87	1.05	96.3	216.05	0.075	0.171	25.83	59.16	0.035	43.40	10.00	9.06
C6	C6- surface	0.82	0.99	170.6	265.60	0.079	0.180	45.29	103.72	0.023	44.80	14.30	8.32
	C6- middle	0.81	0.98	135.7	163.49	0.067	0.154	18.98	43.46	0.030	42.70	8.31	8.56
	C6- bottom	0.81	0.98	161.3	92.10	0.068	0.156	12.77	59.25	0.010	44.40	6.63	8.78
C10	C10- surface	0.79	0.95	245.4	295.79	0.081	0.185	41.08	94.08	0.038	46.20	13.40	8.23
	C10- middle	0.87	1.05	190.5	329.60	0.081	0.184	39.07	89.48	0.044	43.50	11.60	8.46
	C10- bottom	0.84	1.01	178.5	315.16	0.091	0.209	39.39	90.22	0.030	41.90	14.60	8.56
C11	C11- surface	0.98	1.18	215.0	359.10	0.080	0.184	44.99	103.04	0.038	43.10	11.30	8.12
	C11- middle	1.01	1.21	197.7	288.31	0.070	0.160	28.64	65.60	0.016	46.20	5.18	8.26
	C11- bottom	0.96	1.16	211.3	314.62	0.060	0.138	11.17	75.58	0.031	42.30	1.14	8.39
C17	C17- surface	1.20	1.44	234.9	383.14	0.092	0.210	47.69	109.23	0.038	68.30	10.80	8.30
	C17- middle	1.30	1.57	227.6	374.34	0.054	0.123	34.66	79.38	0.031	72.20	12.30	8.31
	C17- bottom	1.37	1.65	206.2	348.45	0.078	0.179	28.98	86.38	0.041	94.30	9.98	8.52
C25	C25- surface	0.79	0.95	289.2	474.02	0.088	0.202	52.50	120.24	0.054	49.00	16.20	8.25
	C25- middle	0.82	0.99	259.8	313.15	0.082	0.189	42.88	98.21	0.013	65.20	16.20	8.36
	C25- bottom	0.76	0.92	199.8	300.74	0.068	0.155	16.11	86.89	0.032	69.80	17.30	8.55
C28	C28- surface	0.80	0.97	231.8	379.38	0.086	0.197	37.26	85.35	0.045	53.90	11.50	8.53
	C28- middle	0.97	1.16	232.1	339.75	0.065	0.149	14.00	52.06	0.026	38.60	12.40	8.69
	C28- bottom	0.91	1.10	238.3	307.19	0.063	0.145	9.99	16.88	0.031	33.40	11.30	8.88
C31	C31- surface	0.91	1.10	201.4	342.71	0.070	0.161	14.00	52.06	0.014	49.70	4.51	8.55
	C31- middle	0.86	1.03	286.9	345.76	0.086	0.197	34.86	79.84	0.017	67.70	5.22	8.46
	C31- bottom	0.91	1.09	228.5	325.35	0.089	0.203	48.09	110.15	0.050	56.10	6.07	8.19
Con 1	Con 1- surface	0.92	1.11	232.6	280.35	0.083	0.131	46.61	106.76	0.053	60.40	16.00	8.60
	Con 1- middle	0.91	1.10	225.7	271.98	0.073	0.138	39.27	59.94	0.054	61.90	17.00	8.71
	Con 1- bottom	0.91	1.10	224.9	271.06	0.072	0.146	34.65	49.36	0.048	87.60	14.90	8.96
Con 2	Con 2- surface	0.87	1.05	252.8	304.62	0.087	0.198	45.69	104.64	0.055	75.40	15.30	8.66
	Con 2- middle	0.81	0.97	237.8	286.58	0.066	0.132	22.38	51.25	0.037	65.90	13.80	8.78
	Con 2- bottom	0.90	1.08	246.9	287.59	0.055	0.127	9.79	22.42	0.020	49.60	13.80	8.91

#### Total nitrogen and alkali-hydrolyzed nitrogen of soil

The total nitrogen content of the soil in the remediation project area ranged from 0.007–0.132% (the mean value was 0.032%), while in the control area it ranged from 0.020–0.055% (the mean value was 0.045%). The total nitrogen content of the soil in this project was found to be at a low level, which was basically consistent with the characteristics of the organic matter content. The content of alkali-hydrolyzed nitrogen was found to be contingent on the content of organic matter content and the amount of nitrogen fertilizer applied. The content of alkali-hydrolyzed nitrogen in the soil exhibited insufficient stability, readily influenced by soil hydrothermal conditions and biological activities. This instability could serve as an indicator of soil’s recent nitrogen supply capacity. The range of soil alkali hydrolyzed nitrogen in different areas of the remediation project was between 31.2 and 94.3 mg/kg, demonstrating significant variability. In the control area, the range was between 49.6 and 87.6 mg/kg.

#### Total phosphorus and readily available phosphorus of soil

The total soil phosphorus (P_2_O_5_) levels in the various plots within the remediation project area ranged from 0.11–0.23% (the mean value was 0.17%), while the total soil phosphorus (P_2_O_5_) levels in the control area ranged from 0.13–0.20% (the mean value was 0.14%). The total phosphorus (P_2_O_5_) content of the soil in this area was found to be at high level comparing the mean values with the experimental group and control group. The subject of this study corresponded to the characteristics of high phosphorus and low nitrogen soil in the Yancheng area.

The readily available phosphorus in the soil was found to be the most effective measure of soil phosphorus accessible for crops. It was also determined to be an important index for evaluating the level of phosphorus supply and soil fertility. The readily available soil phosphorus (P_2_O_5_) levels in the various plots of the remediation project area exhibited significant variability, ranging from 16.8 to 163.9 mg/kg (the mean value was 83.4 mg/kg), while the levels in the control area ranged from 22.4 to 106.8 mg/kg (the mean value was 65.7 mg/kg). In general, the phosphorus levels in the soil in this area were found to be at high when comparing the mean values of the experimental group and to the control group.

#### Total potassium and readily available potassium of soil

The total potassium (K_2_O) content of the soil in the various plots of the remediation project area ranged from 0.59% to 1.65% (the mean value was 1.11%), exhibiting significant variability. In contrast, the total potassium content of the soil in the control area ranged from 0.97% to 1.11% (the mean value was 1.07%). The total potassium content of the soil in this area ranged from Class IV to Class II, with 44% of the samples classified as Class IV, 54% classified as Class III, and 2 samples classified as Class II in Table 4. Consequently, the total potassium content of the soils in this area was found to be at an above average comparing the mean values with the experimental group and control group.

**Table 4:** Classification standard of soil nutrient content.

	1	2	3	4	5	6
Sufficient and lack Analyses	Extremely high	High	Above middle	Middle	Low	Extremely low
Organic matter/(g/kg)	>40	30–40	20–30	10–20	6–10	<6
Total nitrogen/(g/kg)	>2	1.5–2.0	1.0–1.5	0.75–1.00	0.5–0.75	<0.5
Alkali-hydrolyzed nitrogen/(mg/kg)	>150	120–150	90–120	60–90	30–60	<30
Total phosphorus (P_2_O_5_)/(g/kg)	>2	1.5–2.0	1.0–1.5	0.75–1.00	0.5–0.75	<0.5
Available phosphorus (P_2_O_5_)/(mg/kg)	>40	20–40	10–20	5–10	3–5	<3
Total potassium (K_2_O)/(g/kg)	>20	15–20	10–15	5–10	3–5	<3
Available potassium (K_2_O)/(mg/kg)	>200	150–200	100–150	50–100	30–50	<30

Parameters	Mean value	Class
Total potassium	1.02%	III
Available potassium	253.8 mg/kg	I
Total phosphorus	0.18%	II
Available phosphorus	76.7 mg/kg	I
Total nitrogen	0.032%	VI
Alkali-hydrolyzed nitrogen	54.6 mg/kg	V
Organic matter	11.9 g/kg	IV

The content of readily available potassium had been identified as a significant indicator of soil potassium supply and soil fertility. The availability of potassium (K_2_O) in soil exhibited significant variability across different plots of the remediation project, with value ranging from 92.1 to 474.0 mg/kg (the mean value was 313.6 mg/kg). In the control area, soil available potassium levels ranged from 271.1 to 304.6 mg/kg (the mean value was 285.4 mg/kg). As demonstrated in Table 4, the potassium content of soil in this area was found to be range from Class IV to Class I. Specifically, 11% of the samples were classified as Class II, while a substantial majority (84%) was classified as Class I. Consequently, the potassium availability of the soils in this plot was found to be at high level comparing the mean values with the experimental group and control group.

## Discussion

### Evaluation of water quality

Water quality monitoring can be defined as the process of monitoring and measuring the types of pollutants in a water body, the concentration and change trend of various pollutants, and evaluating the state of water quality. As Barcellos & De Souza (2023), Hou et al. (2023) and Yang et al. (2022) had demonstrated, water quality monitoring has the potential to provide the fundamental data required for effective environmental management and ecological remediation.

As stated in the Environmental Quality Standard for Surface Water in China (GB 3838-2002), the water quality of the diversion channel in the wetland farmland remediation project in the dry season was classified as Class III. The mean value of total nitrogen concentration (the main pollutant) was 1.81 mg/L. Furthermore, the mean value of total phosphorus concentration was 0.285 mg/L. The permanganate index and the BOD_5_ were both classified as Class III water, in which the mean value of permanganate index and the BOD_5_ were respectively 5.5 and 4.0 mg/L. The levels of sulphide (<0.005) and heavy metals (<1.0) were found to be relatively low. Consequently, they were all classified as Class I water, with detection values given in Table 1. The water quality during the normal water period was classified as Class III (Thai-Hoang et al., 2022). Additionally, the total phosphorus, total nitrogen and permanganate index levels were all found to be within the parameters of Class III water, the mean values were respectively 0.14, 0.96 and 5.0 mg/L. The levels of sulphide, mercury, lead and cadmium were found to be relatively low and were thus classified as Class I water. In comparison with the low-water period, the arsenic content in the normal-water period exhibited a slightly increase, yet it remained at the level of Class I water as defined in Table 2 (Zeng et al., 2020).

The single-factor evaluation results of the water quality monitoring indicated that the main factors affecting the water quality in the wetland farmland remediation project were total nitrogen, total phosphorus, potassium permanganate index and BOD_5_. The results of the monitoring of the other factors met the Chinese national Class II water quality standards. Therefore, the values of total nitrogen, total phosphorus, potassium permanganate index and BOD_5_ factors were selected in the Nemero Synthesis Index equation, the purpose of which was to calculate the *p* value of the comprehensive pollution index (An et al., 2022). Ultimately, based on a comprehensive evaluation of various parameters, we conclude that the water quality was classified as Class III (moderately polluted).

### Evaluation of bird droppings on soil fertility

The wetland farmland remediation initiative was conceived with the objective of providing a substantial food supplement for migratory birds (Agger et al., 2024). The rice field, which had been sown with wheat, colza oil, Chinese cabbage and other food sources after harvest, was a favored foraging area for wintering migratory birds such as *Grus japonensis* (Kuang et al., 2019). The surrounding ditches also provided drinking water for migratory birds and attracted a significant number of migratory birds, primarily geese, ducks, cranes, and herons (Cital et al., 2022).

The mean organic matter concentration of bird droppings (23.07%) was found to be significantly higher than that of typical soil, as well as considerably higher than that of the soil organic matter content (1.19%) in the area of the wetland farmland remediation project. The accumulation of bird droppings in areas where birds congregated might have some effect on the organic matter content of the soil (Min & Choi, 2022). Concurrently, the mean concentrations of total nitrogen, total phosphorus and total potassium in the wetland farmland remediation project area were detected to be 0.031%, 0.077% and 0.847%, respectively. The mean concentrations of total nitrogen and total phosphorus contents in the bird droppings were found to be 0.246% and 0.336%, which was respectively 7.9 times and 4.8 times higher than those found in the soil of restored farmland. Furthermore, the mean total potassium concentration in the bird droppings (1.173%) was determined to be 1.4 times higher than that of the local soil. The results indicated that the bird droppings might have a certain impact on the nitrogen, phosphorus and potassium content of the soil, thereby changing the fertility of soil. Research had demonstrated that the nitrogen present in bird droppings was capable of volatilizing with ease into the atmosphere, manifesting as NH_3_ in alkaline soil environments (Osada, 2020). Furthermore, the presence of robust nitrification and denitrification removal pathways in saline alkaline soils had been documented (Zhang et al., 2022). As Wang et al. (2021b) demonstrate that the phosphorus in bird droppings readily combined with calcium (Ca) in saline alkaline soil to form stable Ca-P compounds (Ma et al., 2019).

It was estimated that approximately 50,000 birds appeared daily in the wetland farmland remediation project area for approximately 15 days during the winter foraging period (Liu et al., 2022). Subsequent to the termination of the foraging period, an average of approximately 400 birds per day were recorded in the wetland farmland remediation project plot from January to the end of February (a total of 120 days).

As demonstrated in Table 5, the wetland farmland remediation project area had shown an estimated increase in organic matter of approximately 6,629.4 kg, total nitrogen of approximately 97.1 kg, total phosphorus of approximately 75.3 kg, and total potassium of approximately 294.5 kg based on estimated inputs from bird feces. The mean estimated increase per km^2^ was 2.88 kg, 0.042 kg, 0.033 kg, and 0.128 kg, respectively. The estimated increase in organic matter was most significant, followed by total potassium, total nitrogen and total phosphorus (Zhang et al., 2021). Despite the brevity of foraging period following the harvest, the bird density was found to be significantly higher in comparison to the non-foraging period (Brennan, 2020). The wetland farmland remediation project had been shown to provide foraging habitat for birds, the droppings of birds might cause changes in the properties of the soil (Makenova et al., 2024).

**Table 5:** Estimated improvements of soil fertility by bird droppings in the wetland farmland remediation project area.

Fertility index	Increase of fertility/(kg)			Increase of average fertility per km^2^/(kg/km^2^)
	Foraging period	Non-foraging period	Aggregate	
Organic matter	5,894.1	735.3	6,629.4	2.88
Total nitrogen	89.2	7.84	97.1	0.042
Total phosphorus	63.6	11.6	75.3	0.033
Total potassium	257.1	37.4	294.5	0.128

### Evaluation of soil fertility

#### Comprehensive fertility indexes of soil

Soil constitutes the fundamental basis of crop production, with soil fertility representing a comprehensive reflection of all aspects of soil properties. Fertility is typically understood as the ability of the soil to provide and beneficial nutrient conditions and environmental conditions for crop production (Ma et al., 2020; Kumar, Sindhu & Kumar, 2025). The level of soil fertility has a direct impact on the growth of crops, which in turn has a significant effect on the growth and development of soil animals, plants, microorganisms, and even migratory birds associated with crops. Consequently, it is imperative to meticulously examine the fundamental physical and chemical properties of soil and soil fertility within the designated area of wetland farmland remediation projects (Gettys & Moore, 2018; Xie et al., 2019).

Comprehensive standards were consulted in the analysis of sample plots to derive a comprehensive nutrient index. Such an index could be utilized to ascertain the overall fertility of the soil (Sun, Fan & Zhang, 2025; Hernández-Crespo et al., 2016). In the present research, the total nitrogen, alkaline hydrolyzed nitrogen, available phosphorus, and available potassium of the soil were selected for assessment and assigned values. The results were presented in Table 6. The soil fertility in the designated wetland farmland remediation project area was found to be of medium quality, primarily due to a significant deficiency in soil nitrogen content. Subsequent agricultural management and fertilization processes are recommended to appropriately increase the input of organic and nitrogen fertilizers, whilst concomitantly controlling input of phosphate and potassium fertilizers (Zheng et al., 2022).

**Table 6:** Comprehensive fertility indexes of soil in the wetland farmland remediation project area and control area. Reference comprehensive fertility and soil fertility grade: 0–40: Low; 41–80: Middle; 81–100: High.

Comprehensive fertility index		*I*	Fertility degree
The farmland remediation project area	Total nitrogen	57	Middle
	Alkali-hydrolyzed nitrogen	62	Middle
The control area	Total nitrogen	63	Middle
	Alkali-hydrolyzed nitrogen	73	Middle

As posited by Ma et al. (2020), the analysis of existing literature suggested that the process of forming a substantial quantity of Ca-P and accumulating it within the soil was facilitated by the absorption of exogenous phosphorus (as a fertilizer) in an alkaline environment proximate to the sea. Furthermore, the presence of significant avian populations within the region suggests the potential for partial phosphorus input *via* bird excrement (Jauffrais et al., 2015; Zhang et al., 2021; see also Table 5). Because plants absorbed relatively little phosphorus, phosphorus accumulated in the soil of this region due to the absence of any alternative means of removal. However, the predominant source of nitrogen was from biological processes associated with plant life, yet the region’s plant population was sparse. Furthermore, the presence of robust nitrification and denitrification removal pathways in the soil has been demonstrated, resulting in the observation of relatively low nitrogen content in the region’s soil (Li, Zhang & Shi, 2023).

#### Fertility distribution of soil

**Figure 4:**
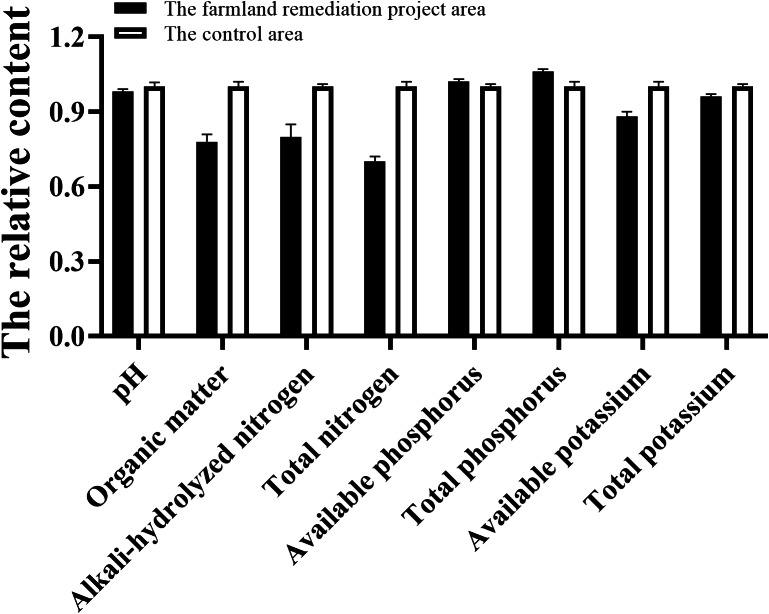
Comparison of soil nutrients between the wetland farmland remediation project area and control area. *: *P* ≤ 0.05; ns: *P* > 0.05.

As demonstrated in Fig. 4, a comparison was made of soil nutrients in the wetland farmland remediation project area and the control area. All nutrient indices were subjected to transformation in order to facilitate cartographic representation and comparative analysis. pH, organic matter, total nitrogen, alkaline hydrolyzed nitrogen, readily available potassium and total potassium were all found to be less than those in the control area, while the total phosphorus and readily available phosphorus were both found to be little greater than the levels in the control area. Meanwhile, the content of total nitrogen in the farmland remediation project area was significantly higher than that in control area (*p* < 0.05). Given the similarity in soil material, the observed difference might be primarily attributable to the varying absorption capabilities of the plants and the effect of rainwater runoff (Schurkamp, Lishawa & Ohsowski, 2024). Furthermore, the substantial avian congregation observed within the wetland farmland remediation area might prove to be a factor in the process of soil phosphorus changing within this particular ecosystem.

**Figure 5:**
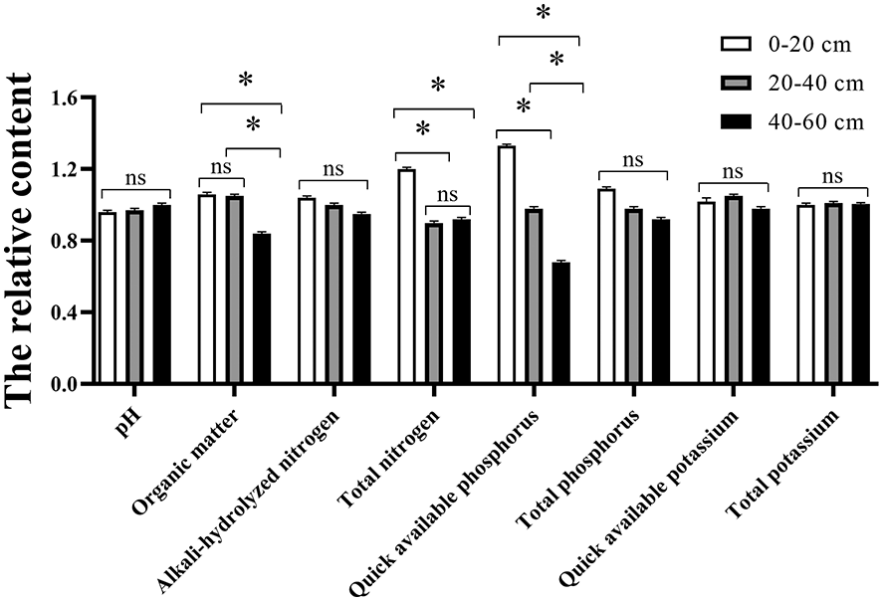
The vertical distribution of soil nutrients in the wetland farmland remediation project area. *: *P* ≤ 0.05; ns: *P* > 0.05.

The vertical distributions of nutrients within the wetland farmland remediation area had been demonstrated in Fig. 5. There were significant differences in the organic matter, total nitrogen, and readily available phosphorus among different soil layers (*p* < 0.05). As shown in Fig. 5, with the exception of pH, the content of other soil fertility indices was high in the surface layer and low in the bottom layer, thereby indicating that soil fertility was predominantly concentrated in the surface layer (Zamorano et al., 2018). As the soil layer deepened, there was a gradual decrease in soil fertility, which may be primarily attributable to the input of exogenous fertilizer (including fertilizer and bird droppings) (An et al., 2025). The pH exhibited a low value in the surface layer and a high value in the bottom layer, this phenomenon was attributed to the downward leaching of Cl^−^ and OH^−^ in saline alkaline soil by rainfall (Pipil, Haritash & Reddy, 2022). Therefore, considering multiple parameters and multiple layers of soil, the soil is classified as Class III (moderately polluted).

### Prospects and suggestions

The present study revealed that by planting different crops, the local water quality and soil properties could be improved, which would attract more winter migratory birds to the Yancheng Wetland, providing a guarantee for the ecological protection of the Yancheng Wetland. Based on this research, we would investigate whether the feeding and defecation behaviors of birds really have an enhancing effect on the soil fertility in this area in the future. If so, which parameters would be positively affected? Furthermore, in the wetland farmland remediation project, the water quality was classified as category III. The main pollutants were total nitrogen and BOD_5_. The sources of these main pollutants should be further investigated.

In the subsequent soil improvement process, it was recommended to further enhance soil desalination, dealkalization, water replenishment; increase organic matter (organic fertilizers); and appropriately retain some plant residues and fallen materials. At the same time, further reducing human interference would improve food sources for overwintering birds. Furthermore, based on the characteristics of the local soil fertility, it was suggested that the fertilization method should be appropriately changed in future planting and field management. The focus should be on supplementing nitrogen fertilizer and organic matter, while the application of phosphate and potassium fertilizers could be appropriately controlled.

## Conclusion

The present paper demonstrated that the implementation of the wetland farmland remediation initiative results in a number of notable environmental benefits. These benefits included the enhancement of water quality and soil fertility within the wetland ecosystem, the improvement of the surrounding ecological environment, an increase in the number of migratory birds that chose to reside in the area, and an overall enhancement of the capacity of the Yancheng Wetland for the protection of rare bird species. The water pollutants present in the remediation area at different periods were primarily total nitrogen and BOD_5_, which were classified as category III and belonged to the state of moderate pollution in the wetland farmland remediation area. The soil comprehensive fertility index of the wetland farmland remediation area was medium, primarily exhibiting the characteristics of low nitrogen, low organic matter, high phosphorus and high potassium. The organic matter, nitrogen, phosphorus and potassium levels in the soil might be enhanced by the fertilizers and bird droppings. The soil fertility was comprehensively categorized as Class III (moderately polluted). Following a comprehensive analysis of the water quality and soil fertility in this area, it was recommended that changes be made to the fertilization and field management practices. The suggested modification to the fertilization method for the future planting included the addition of nitrogen and organic matter, in addition to the precise regulation of phosphate and potassium fertilization. Subsequent wetland farmland remediation projects can continue to enhance the water quality and soil quality, whilst simultaneously enriching the biodiversity of the area and attracting an increasing number of wintering migratory birds to the wetland.

## Supplemental Information

10.7717/peerj.20406/supp-1 Supplemental Information 1 Supplementary Material

10.7717/peerj.20406/supp-2 Supplemental Information 2 Figure data

## Additional Information and Declarations

### Data Availability

The following information was supplied regarding data availability: This data is available in the Supplemental File.
