# Supplementary material for: Evaluation of water quality and soil fertility in remediated farmland for protection of wetland ecology by planting different crops
Source: PeerJ. 2025 Nov 28;13:e20406. doi: 10.7717/peerj.20406 (PMC12667689; doi:10.7717/peerj.20406)
Supplement: Supplemental Information 1 [file peerj-13-20406-s001.docx]

**Evaluation of water quality and soil fertility in remediated farmland for protection of wetland ecology by planting different crops**

Changqing Liu^a^, Zhongxiang Sun^a^, Hongyang Wang^a^, Tianwen Chen^b^, Lina Deng^a^, Li Zhang^a^, Huixing Liang^a^, Yuxiang Shen^a^, Hongshan Li^a*^, Hai Cheng^c*^

a College of Marine and Biological Engineering, Yancheng Institute of Technology, Yancheng, Jiangsu, PR China

b Business School, Yancheng Polytechnic College, Yancheng, Jiangsu, PR China

c Jiangsu Yancheng National Rare Bird Nature Reserve Management Office, Yancheng, Jiangsu, PR China

***Correspondence author**

Dr. Hongshan Li

Schoole of Marine and Biological Engineering, Yancheng Institute of Technology

No. 211 Jianjun East Road, Yancheng, Jiangsu 224051, People’s Republic of China

E-mail: Lihongshan202303@163.com

Tel: +86-13705105217

Mr. Hai Cheng

Jiangsu Yancheng National Rare Bird Nature Reserve Management Office

No.16 Xinyang Port Wanghe Road, Huangjian Town, Tinghu District, Yancheng City, Jiangsu Province, People’s Republic of China

E-mail: chenghai202301@163.com

Tel: +0515-89211136

**Table legend**

**Table. S1** The survey results of migratory birds in different periods (R: Remediated area, C: Control area).

**Figure legend**

**Fig. S1** The latitude and longitude location of wetland farmland remediation area.

**Table. S1** The survey results of migratory birds in different periods (R: Restored area, C: Control area).

| Species | Nov.15 | | Nov.16 | | Nov.17 | | | Nov.18 | | Nov.22 | | Nov.26 | | Nov.27 | | Nov.28 | | Nov.29 | | Dec.14 | | Dec.18 | |
| --- | --- | --- | --- | --- | --- | --- | --- | --- | --- | --- | --- | --- | --- | --- | --- | --- | --- | --- | --- | --- | --- | --- | --- |
|  | R | C | R | C | | R | C | R | C | R | C | R | C | R | C | R | C | R | C | R | C | R | C |
| *Ardea cinerea* | / | / | 2 | / | | / | 1 | / | / | / | 1 | 1 | / | 1 | / | 2 | / | / | / | 10 | / | 7 | 1 |
| *Egretta garzetta* | / | / | / | / | | / | / | / | / | / | / | / | / | 1 | / | / | / | / | / | 20 | / | / | / |
| *Anser fabalis* | 6315 | 1220 | / | 1481 | | / | 1100 | / | 10 | 41 | 55 | / | / | / | 170 | 7 | 66 | 4 | 285 | 606 | 1706 | / | / |
| *Cygnus columbianus* | / | / | / | / | | / | / | / | / | / | / | / | / | / | / | 3 | / | 4 | / | / | / | / | / |
| *Anas acuta* | / | / | / | / | | / | / | / | / | 42 | 30 | 4000 | / | 650 | / | / | / | / | / | / | / | / | / |
| *Anas platyrhynchos* | / | / | / | 200 | | 2500 | / | 400 | / | 13110 | 120 | 56000 | / | 16600 | / | / | / | 14000 | / | / | / | / | / |
| *Anas poecilorhyncha* | / | / | 5000 | 500 | | 3950 | / | 600 | / | 2640 | 160 | 20000 | / | 3750 | / | / | / | 5000 | / | / | / | / | / |
| *Anas strepera* | / | / | / | / | | / | / | / | / | / | / | / | / | / | / | / | / | 1000 | / | / | / | / | / |
| *Elanus caeruleus* | / | / | / | / | | / | / | / | / | / | / | / | / | / | / | / | / | / | / | / | / | / | 1 |
| *Phasanus colchicus* | 2 | / | / | / | | 3 | / | 2 | / | 1 | / | 3 | / | / | / | / | / | / | / | / | / | 4 | 9 |
| *Grus grus* | 66 | / | / | 17 | | 4 | 28 | / | / | 50 | 56 | 22 | / | 25 | 55 | 20 | 35 | 22 | 79 | 17 | 24 | 51 | 16 |
| *Grus monacha* | 4 | / | / | / | | / | 6 | / | / | 2 | / | / | / | / | 2 | / | 6 | 3 | 5 | 4 | 3 | / | / |
| *Grus japonensis* | / | / | / | / | | / | 13 | / | 3 | / | 8 | 24 | / | 11 | 9 | 4 | / | 4 | / | 5 | / | 12 | 2 |
| *Grus vipio* | / | / | / | / | | / | / | / | / | / | / | 1 | / | 1 | / | / | / | / | / | 4 | / | 5 | / |
| *Streptopelia chinensis* | / | / | / | / | | / | / | / | / | / | / | 10 | / | / | / | / | / | / | / | / | / | / | / |
| *Lanius schach* | 1 | / | / | / | | / | / | / | / | / | / | / | / | / | / | / | / | / | / | / | / | / | / |
| *Acridotheres cristatellus* | 1 | / | / | / | | / | / | / | / | / | / | / | / | / | / | / | / | / | / | / | / | / | / |
| *Pica pica* | 30 | / | / | / | | 9 | / | / | / | 10 | / | 45 | / | / | / | / | / | / | / | / | / | / | / |
| Total | 6419 | 1220 | 5002 | 2198 | | 6466 | 1148 | 1002 | 13 | 15896 | 430 | 80106 | / | 21039 | 236 | 36 | 107 | 20037 | 369 | 666 | 1733 | 79 | 29 |


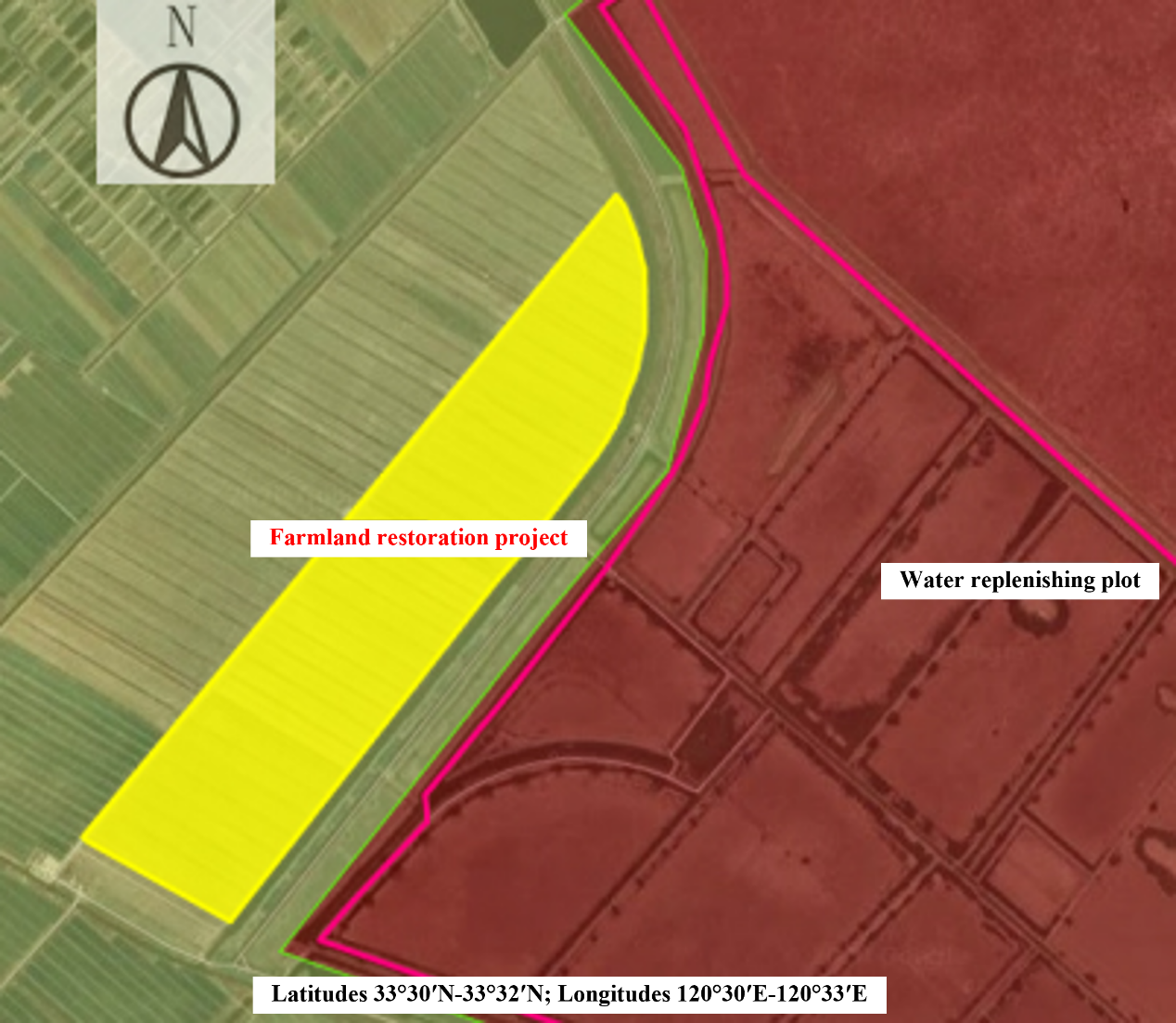


**Fig. S1**
